# Exploring Celiac Disease: A Case Analysis of Multi-Systemic Symptoms and Effective Dietary Intervention in a Young Female

**DOI:** 10.7759/cureus.43839

**Published:** 2023-08-21

**Authors:** Mahmoud Ahmed, Vyshnavidevi Sunkara, Muhammad S Khan, Maryam Affaf, Mohammad Ahsan Anwaar

**Affiliations:** 1 Internal Medicine, St George University, New York, USA; 2 Medicine, Katuri Medical College and Hospital, Guntur, IND; 3 Internal Medicine, Foundation University Medical College, Islamabad, PAK; 4 Internal Medicine, Women Medical College, Abbottabad, PAK; 5 Internal Medicine, CMH Lahore Medical College and Institute of Dentistry, Lahore, PAK

**Keywords:** iron deficiency anemia (ida), intraepithelial lymphocytes, dermatitis herpetiformis, ttg-iga antibodies, celiac disease (cd)

## Abstract

This study presents the case of a 23-year-old woman diagnosed with celiac disease (CD), a condition triggered by an immune response to gluten, leading to inflammation in the small intestine. The patient manifested typical gastrointestinal symptoms, including diarrhea, abdominal pain, and vomiting, complemented by extra-intestinal signs such as fatigue and skin rashes. Diagnosis was corroborated through the presence of tTG-IgA antibodies and distinct histological changes in the duodenum. A notable finding was the patient's iron deficiency anemia, directly linked to the duodenal damage caused by CD. Effective management, encompassing a strict gluten-free diet and iron supplementation, resulted in marked improvement in her condition. This case accentuates the significance of early CD detection, especially in patients exhibiting a combination of gastrointestinal and extra-intestinal symptoms. Emphasis is placed on the pivotal role of timely diagnosis, adherence to a gluten-free regimen, and sustained monitoring to ensure patient well-being and prevent complications.

## Introduction

Gluten and related proteins can trigger sensitivities leading to celiac disease (CD), a condition marked by immune-driven inflammation of the small intestine [1]. This autoimmune response against the duodenal brush border, due to dietary gluten sensitivity, results in malabsorption and manifests in both gastrointestinal and extra-intestinal symptoms [2]. Globally, the prevalence of celiac disease is estimated to be between 0.5% and 1% [3]. Recent studies emphasize the role of intestinal intraepithelial lymphocytes and cyclic nucleotides in causing villous atrophy [4]. Although CD is often considered rare among individuals of African American, Jewish, and Mediterranean descent, emerging evidence suggests racial and ethnic predispositions to the disease [4]. This case report is pivotal as it underscores the multifaceted presentation of CD, emphasizing the importance of early detection and comprehensive management. Through this report, we aim to highlight the nuanced manifestations of CD, emphasizing the significance of recognizing both typical and atypical symptoms for timely intervention.

## Case presentation

A 23-year-old female presented with a constellation of symptoms, including severe diarrhea, lower abdominal pain, vomiting, fever, fatigue, and rashes on her hands and feet. Historically, she had been in good health until seven years prior, when she began experiencing intense diarrhea. This was accompanied by significant abdominal pain, vomiting, fever, and generalized fatigue. Notably, the onset of diarrhea was triggered each time she consumed wheat bread, while milk consumption did not produce a similar effect. The fecal matter was pale, devoid of blood or any foul odor. The abdominal pain was persistent, localized to the lower abdomen, and alleviated only with medication. She experienced frequent bouts of vomiting, up to four to five times a day, characterized by a yellowish hue without any associated foul smell or chest discomfort. The fever, fluctuating between 100 and 101°F, was more pronounced in the mornings and evenings and was exacerbated by regular meals, which also triggered diarrhea. Distinct rashes appeared on her hands and feet, which were neither painful nor discharging.

Despite attempting home remedies, her condition deteriorated. She became severely anemic, necessitating a blood transfusion. On her own accord, she eliminated wheat bread from her diet and observed an initial improvement. However, her symptoms resurfaced within weeks. Six years ago, she was diagnosed and treated for jaundice. As her health continued to decline, she was referred to a tertiary care hospital for a comprehensive evaluation.

**Figure 1:**
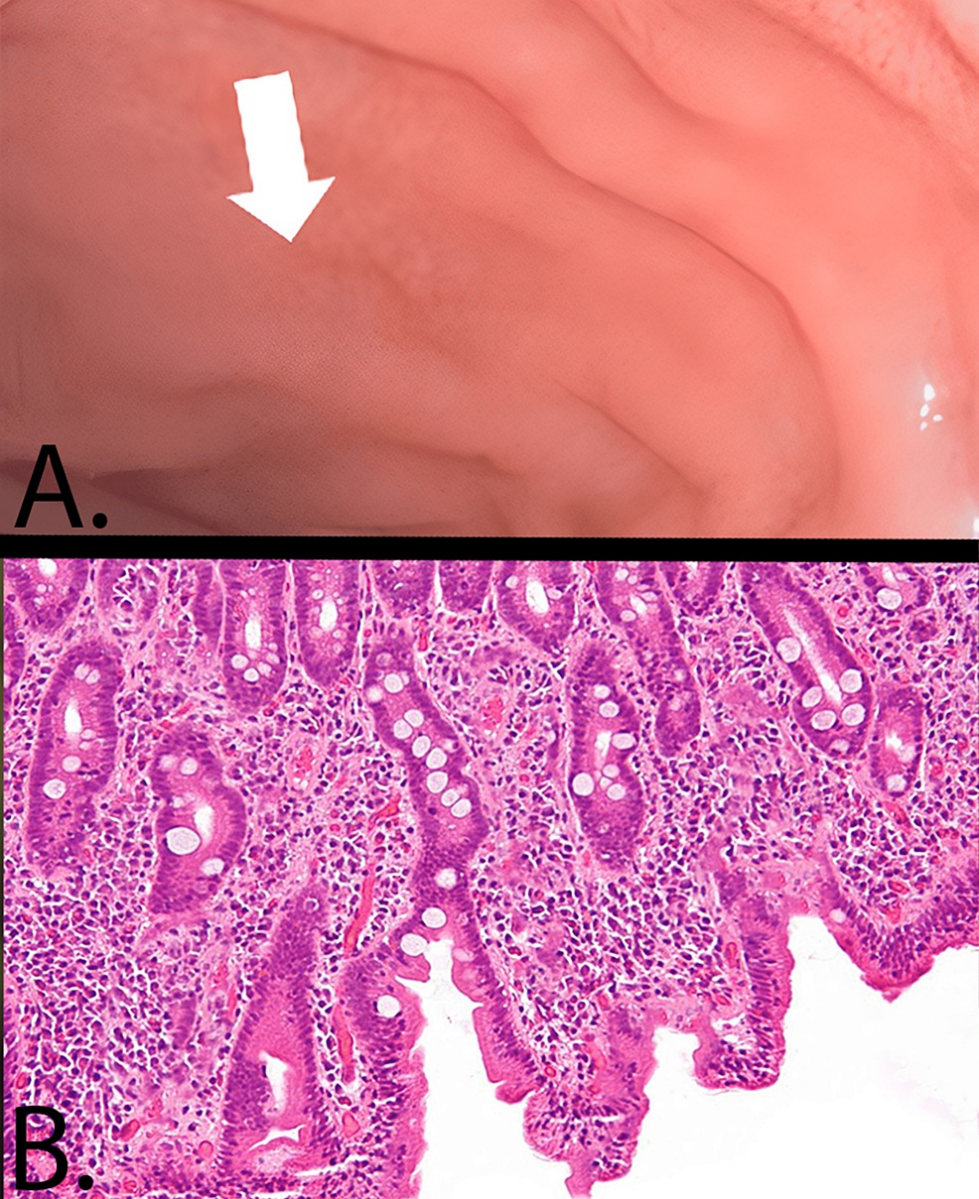
(A) Duodenal endoscopy shows reduced fold height in the second part of the duodenum. (B) Histopathological investigations (hematoxylin and eosin-stained duodenal slide at 40x magnification) show an increased presence of intraepithelial T-cell lymphocytes.

Diagnostic tests revealed positive tTG-IgA antibodies. Further investigations, including endoscopy and histopathological examination (Figure 1) of the gut, indicated reduced fold height in the second part of the duodenum and an increased presence of intraepithelial T-cell lymphocytes. These findings confirmed a diagnosis of CD. Baseline investigations are elaborated in Table 1.

**Table 1:** Complete blood work-up. INR: international normalized ratio, APTT: activated partial thromboplastin time, WBC count: white blood cells count, RBC: red blood cells, HCT: hematocrit, MCV: mean corpuscular volume, MCH: mean corpuscular hemoglobin, MCHC: mean corpuscular hemoglobin concentration, TIBC: total iron binding capacity, * : showing derangement

Coagulation Profile	Results	Reference range
Prothrombin Time-Control	11	10-14 seconds
Prothrombin Time-Patient	12	Up to 13 seconds
INR	1	0.9-1.3
Control Time	26	25-35 seconds
APTT	30	Up to 31 seconds
Hemogram		
WBC count	2.92*	4-11 x10^9^/L
Total RBC	3.740*	3.8-5.2 x10^12^/l
Hemoglobin	8.3*	13-18 (g/dL)
HCT	28.44*	35-46%
MCV	84.82*	77-95 fl
MCH	20.8*	26-32 (pg)
MCHC	29.8*	32-36 (g/dL)
Platelets	196	150-400 x10^9^/L
Neutrophils	47.4	40-80%
Lymphocytes	50.3*	20-40%
Special Chemistry		
Serum total calcium	9.1	8.1-10.4 mg/dl
Serum electrolytes	3.5	0.4-5.0mIU/l
TIBC	410	228-428ug/dl

The patient's management included a gluten-free diet, avoiding items such as wheat bread, cakes, sweets, biscuits, and certain juices. Given her anemia and the upper limit total iron binding capacity (TIBC), she was prescribed iron supplements. Her health improved significantly post-treatment.

However, a few weeks ago, she returned with similar complaints. The recurrence of symptoms was attributed to accidental wheat consumption. Recurrence of symptoms that are stated above resulted in less than 12 hours after accidental wheat bread consumption. She was treated with fluid replacement therapy and analgesics. Currently, the patient is in a stable and healthy condition.

## Discussion

Since its identification in the 1950s, the diagnosis of celiac disease has seen a significant rise, especially with the widespread adoption of jejunal biopsies. The incidence of this condition ranges from 12/100,000 to 300/100,000, with a higher prevalence in females compared to males. However, these figures primarily represent typical cases [4]. While celiac disease is more prevalent in African American populations, it is less common in Asian countries. Estimating the true prevalence of this disease in any demographic is challenging due to the presence of atypical and asymptomatic cases, especially among adults [4].

The patient's gastrointestinal issues, rashes, and stunted growth led to multiple differential diagnoses, including inflammatory bowel disease (IBD), irritable bowel syndrome (IBS), food allergies, and infectious gastroenteritis. Giardiasis and small intestinal bacterial overgrowth (SIBO) were also considered. Yet, the blend of clinical, serological, and histopathological findings confirmed celiac disease, emphasizing the value of thorough differential diagnosis.

The patient was diagnosed with celiac disease due to positive tTG-IgA antibodies and notable histopathological changes in the duodenum, including reduced duodenal fold height. She had a severe gluten intolerance, leading to immediate vomiting and diarrhea after gluten intake. The compromised proximal duodenum, vital for iron absorption, resulted in iron deficiency anemia (IDA) - a common extra-intestinal manifestation of celiac disease with a prevalence of 12% to 82% [5]. Her anemia, exacerbated by significant iron loss, required a blood transfusion. She's now on iron supplements to counteract this deficiency.

One of the striking features of this case was the patient's acute and pronounced intolerance to gluten. While many individuals with celiac disease experience symptoms following gluten ingestion, the immediacy and severity of the patient's reactions were noteworthy. Within a short time frame post-consumption of gluten-rich meals, she experienced intense episodes of vomiting and diarrhea. Such an immediate and severe response suggests a heightened sensitivity to gluten, emphasizing the critical importance of dietary vigilance for such patients. This rapid onset of symptoms can be particularly challenging for patients, as even minor dietary missteps can lead to significant discomfort and health risks. Clinicians should be aware of the potential for such acute reactions in some CD patients and provide thorough dietary guidance and education to prevent inadvertent gluten exposure.

Dermatitis herpetiformis, marked by itchy eruptions and burning blisters, results from an immune response to gluten sensitivity. Though it mimics herpes, it's unrelated to the herpes virus. Those with celiac disease, like our patient who had rashes on her extremities, can exhibit this. A gluten-free diet and targeted medications [6] have significantly improved her skin condition.

Beyond typical gastrointestinal symptoms, the patient displayed growth retardation, an extra-intestinal manifestation of celiac disease. This growth delay, more prevalent in pediatric CD cases, highlights the disease's systemic effects. In adults, it signals extended malabsorption. Such retardation, primarily due to nutritional deficiencies and exacerbated by consistent vomiting and diarrhea, can sometimes be the sole diagnostic symptom. However, a gluten-free diet often leads to marked growth improvement [7]. This case underscores celiac disease's broad systemic consequences and the importance of holistic care addressing all manifestations. Presently, with a strict gluten-free diet and iron supplementation, the patient's condition remains stable and well-managed.

## Conclusions

This case report underscores the multifaceted and often underrecognized manifestations of celiac disease. The acute and pronounced intolerance to gluten exhibited by the patient, coupled with the immediate onset of severe symptoms, highlights the spectrum of gluten sensitivity and the challenges faced by patients in managing their condition. Furthermore, the presence of growth retardation as an extra-intestinal manifestation in an adult patient serves as a poignant reminder of the systemic reach of CD beyond the gastrointestinal domain. Such manifestations, especially when they are the sole presenting symptoms, can complicate the diagnostic process and delay timely intervention. This case accentuates the importance of a comprehensive clinical evaluation, timely diagnosis, and rigorous patient education. It serves as a testament to the significance of recognizing both typical and atypical presentations of CD, emphasizing the need for heightened clinical vigilance. The findings presented herein not only contribute to the growing body of literature on CD but also underscore the imperative of early detection and intervention to ensure optimal patient outcomes.

## References

[1] de Almeida Menezes M, Cabral V, Silva Lorena SL (2017). Celiac crisis in adults: a case report and review of the literature focusing in the prevention of refeeding syndrome. Rev Esp Enferm Dig.

[2] Murphy AK, Norton JA, Pflederer BR (2020). Celiac disease in an adult presenting as behavioral disturbances. Am J Case Rep.

[3] Kravchuk S, Mishchanchuk V, Kozyk M, Strubchevska K (2023). A clinical case of a celiac crisis in an adult with type 1 diabetes and neurological symptoms. Cureus.

[4] Ahluwalia ML, Larbi EB, Fadali GA (1996). Adult celiac disease: report of a case. Ann Saudi Med.

[5] Talarico V, Giancotti L, Mazza GA, Miniero R, Bertini M (2021). Iron deficiency anemia in celiac disease. Nutrients.

[6] Salmi TT (2019). Dermatitis herpetiformis. Clin Exp Dermatol.

[7] Nemet D, Raz A, Zifman E, Morag H, Eliakim A (2009). Short stature, celiac disease and growth hormone deficiency. J Pediatr Endocrinol Metab.

